# Retraction: MicroRNA-34A inhibits the growth, invasion and metastasis of gastric cancer by targeting PDGFR and MET expression

**DOI:** 10.1042/BSR-2014-0020_RET

**Published:** 2023-04-21

**Keywords:** gastric cancer, MET, miR-34a, PDGFR

This Retraction follows an Expression of Concern relating to this article previously published by Portland Press. This article is being retracted from *Bioscience Reports* at the request of the Editor-in-Chief and the Editorial Board following receipt of a notification from a reader, alerting the Editorial Board to various concerns within the manuscript:

- In Figure 5C, sections of multiple panels appear within other panels (either in entirety or partially) from Figure 3I of Peng et al. (2014) (doi: 10.1371/journal.pone.0094422). We note that these papers also have the same first and corresponding authors, are of similar subject matter, and that similarities have been identified between the texts.
- In Figure 5C, a section of the Migration/cont miR panel and the Invasion/miR34a+MET+ panel appear as the Figure 6E panels (both rotated 90 degrees) of Jiang et al. 2017 (doi: 10.3892/or.2017.5641).
- The Figure 1B miR-29b/GAPDH bands seem to appear (rotated 180 degree) in the last two GAPDH bands of Figure 5A.
- In the Figure 4F cell panels, a section of the Migration/PDGFRa/B-miR34a- panel seem to also appear in the Invasion PDGFRa/B+miR34- panel.
- In Figure 5C, part of the Migration/contmiR panel seems to also appear in the Migration miR34a+PDGFRa/B+MET+ panel, as well as part of the Invasion/contmiR panel appearing as duplicated in the Invasion miR34a+PDGFRa/B+MET+panel.

The authors have been contacted with regards to the retraction and have not responded to the Journal's queries or the concerns raised. Given the extent of the issues raised, the Editorial Board stand by the decision to retract the article.

